# Breaking Apterism in *Elytrosphaera* Blanchard 1845: New Synonymy, Generic Transfer, Redescription of the Only Winged Species, *Grammodesma luridipennis* (Baly 1859) (Coleoptera: Chrysomelidae: Chrysomelinae), with Comparative Morphological Comments

**DOI:** 10.1007/s13744-026-01434-2

**Published:** 2026-09-10

**Authors:** Aline Sampaio, José Albertino Rafael

**Affiliations:** 1 Programa de Pós–Graduação em Ciências Biológicas (Entomologia), Instituto Nacional de Pesquisas da Amazônia (INPA), Manaus, Amazonas Brazil; 2 Facultad de Agronomía, Museo del Instituto de Zoología Agrícola Francisco Fernández Yépez (MIZA), Universidad Central de Venezuela, Aragua, Venezuela

**Keywords:** Atlantic forest, Brazil, Chrysomeloidea, Morphology, Taxonomy

## Abstract

In this study, we transfer *Grammodesma luridipennis* (Baly 1859) to *Elytrosphaera* Blanchard 1845 and redescribe the species. *Elytrosphaera lahtivirtai* Bechyně, 1951 is proposed as a **new junior synonym** of *E. luridipennis*. Hind wings are documented for the first time in *E. luridipennis*, making it the only species of *Elytrosphaera* currently known to be winged. Male and female genitalia are described for the first time. We provide morphological and distributional data for *E. luridipennis*, including external morphology and wing venation. In addition, we compare the morphology of *Grammodesma* and *Elytrosphaera*, highlighting diagnostic characters that distinguish the two genera, and distinguish *E. luridipennis* from *Grammodesma rubroaenea* (Stål, 1859), one of the *Grammodesma* species most similar to *E. luridipennis* in coloration. We also compare *E. luridipennis* with *Elytrosphaera xanthoptera xanthoptera* (Gistel, 1848), highlighting diagnostic differences between these taxa.

## Introduction

*Grammodesma* Achard 1923 is a South American genus comprising 31 species occurring in Bolivia (Cochabamba) and Brazil, of which 30 are recorded from the latter country. For most species, the distribution data are imprecise, with Brazil often cited only at the national level as the locality of occurrence. Among the species recorded from Brazil, those for which detailed distributional information is available occur in the Northeastern region (Bahia and Pernambuco), where the Caatinga, Cerrado, and Atlantic Forest biomes are present, and in the Southeastern region (Espírito Santo, Minas Gerais, Rio de Janeiro, Santa Catarina, and São Paulo), where the Atlantic Forest predominates. It should be noted that Minas Gerais also includes portions of the Caatinga and Cerrado. It is worth emphasizing that the Atlantic Forest is the biome shared among all Brazilian states in which the genus occurs (Bechyně, 1952; Sekerka & Sampaio 2025).

One of the species attributed to *Grammodesma* in Brazil, *Grammodesma luridipennis* (Baly 1859), which has been reported in the literature from two states in the southeastern region (Espírito Santo and Minas Gerais), is not conform the diagnostic characteristics of *Grammodesma*, which is characterized, among other features, by a projected base of the prosternal process. Instead, it corresponds to *Elytrosphaera* Blanchard 1845, in which the base of the prosternal process is not projected, among other distinguishing traits that will be discussed in detail below. *Elytrosphaera* is likewise a South American genus, currently comprising eight species recorded from Brazil, all endemic to the Atlantic Forest and occurring in the southeastern and southern regions of the country. Only a single subspecies, *Elytrosphaera vittata misionea* Bechyně, 1950, is known to occur in Argentina (Misiones).

Within *Elytrosphaera*, *Elytrosphaera lahtivirtai* Bechyně, 1951 has long been regarded as a morphologically distinctive species; however, it exhibits all diagnostic characters of *Grammodesma luridipennis*. Despite this clear correspondence, both taxa have historically been treated as separate species. At the time of its description, *E. lahtivirtai* was not compared with *G. luridipennis*, but instead with *Elytrosphaera xanthoptera xanthoptera* (Gistel, 1848), a species that is clearly distinct from *E. lahtivirtai*.

In light of these circumstances, the present study establishes *Elytrosphaera lahtivirtai* Bechyně, 1951 as a **new junior synonym** of *Grammodesma luridipennis* (Baly 1859) and transfers the latter to *Elytrosphaera* Blanchard 1845. *Elytrosphaera luridipennis* Baly 1859 is redescribed, and the male and female genitalia are described for the first time. We also provide photographs of the external morphology, including the wings with labeled venation, and the genitalia, as well as new distributional data for the species. In addition, a comparative morphological table (Table 1) is provided to distinguish *E. luridipennis* from *Grammodesma rubroaenea* (Stål, 1859), one of the *Grammodesma* species most similar to *E. luridipennis* in coloration, and to present diagnostic characters for both genera. A second comparative table (Table 2) is provided to differentiate *E. luridipennis* from *E. xanthoptera xanthoptera* (Gistel, 1848).

## Material and methods

The illustrations and measurements were carried out at the Instituto Nacional de Pesquisas da Amazônia (INPA) using a Leica M205A stereomicroscope equipped with a Leica DMC4500 digital camera. Image adjustments and plate assembly were performed using Adobe Photoshop CS2 software. The terminology adopted for external morphology follows Lawrence et al. (2010). For the male genitalia, the terminology follow that proosed by Bezděk et al. (2012), Rodrigues & Mermudes (2015), and Vela & Daccordi (2021). For the female genitalia, the terminology follow Konstantinov (1998), Daccordi et al. (2020), and Bezděk (2015). Wing venation terminology follows Jolivet (1957), in which C = costa, Sc = subcosta, R1 = radial vein (first radial), M1 = anterior median vein, M2 = posterior median vein, Cu = cubital vein, A1 = first anal vein, A2 = second anal vein, r = radial crossvein, rm = radial-median crossvein, m = median crossvein, j = median–cubital junction (junction vein), rt = radial triangle, ap = apertum (median cell), an = anal cell, and M1a = accessory median vein.

The endophallus of pinned specimens was everted and inflated with 70°GL (Gay-Lussac degrees, corresponding to 62.4° INPM, Instituto Nacional de Pesos e Medidas) gel alcohol using an insulin syringe through the basal ostium of the aedeagus (basal lobe).

Geographical distribution data were obtained from specimen labels, the relevant literature, and iNaturalist.

Type and non-type specimens were examined either directly or through photographs provided by the following collections (curators in parentheses). The examined material included type specimens of *E. luridipennis*, *E. lahtivirtai*, and *G. rubroaenea*, as well as non-type specimens of *E. luridipennis* (64 specimens) and *E. x. xanthoptera* (14 specimens):

- **CEIOC** – Coleção Entomológica do Instituto Oswaldo Cruz, Rio de Janeiro, Brazil (Márcio Felix and Cláudia Leal).
- **DZUP** – Coleção Entomológica Pe. Jesus Santiago Moura, Universidade Federal do Paraná, Curitiba, Brazil (Lúcia Massutti and Norma Ganho).
- **LUOMUS** – Finnish Museum of Natural History, University of Helsinki, Helsinki, Finland (Jaakko Mattila, Pekka Malinen and Sergei Tarasov).
- **MIZA** – Museo del Instituto de Zoología Agrícola Francisco Fernández Yépez, Maracay, Venezuela (Vilma Savini and Quintin Arias).
- **MZSP** – Museu de Zoologia da Universidade de São Paulo, São Paulo, Brazil (Sônia Casari and Michele Leocadio).
- **NHMUK** – Natural History Museum, London, United Kingdom (Max Barclay, Michael Geiser and Keita Matsumoto).
- **NHRS** – Naturhistoriska Riksmuseet, Stockholm, Sweden (Johannes Bergsten and Anna Jerve).

## Taxonomy

***Grammodesma*** **Achard 1923**

*Grammodesma* was originally established to accommodate a group of species previously assigned by Stål (1862) to the 24th division of his monograph. Over time, this taxon has frequently been confused with *Desmogramma* Erichson, 1847, due to several shared morphological traits. However, Achard (1923) highlighted important diagnostic differences in the original description of the genus, thus supporting its taxonomic distinction:

“These species exhibit, as in *Desmogramma*, a prosternum that is strongly elevated and abruptly depressed anteriorly, as well as a pronotum with subparallel margins extending beyond the midpoint and then abruptly constricted into an arched outline. The anterior angle is produced, slightly mucronate, and the pore is situated on this mucro, well outside the marginal groove. However, they differ by their more robust body form, larger size, distinct punctation and coloration patterns, and by the presence, on the male anal segment, of a longitudinal pit that is generally deep.”

However, the last two diagnostic characters proposed by Achard (1923) to distinguish *Grammodesma* from *Desmogramma* may not be sufficiently reliable for separating the genera. The first concerns the pattern of punctation and coloration. In both *Desmogramma* and *Grammodesma*, the punctation follows essentially the same pattern, being diffuse on the head and pronotum in both genera. However, in *Grammodesma* the elytral punctation may be coarse or fine and arranged in well-defined or poorly delimited rows, particularly along the outer margins and at the apex. In contrast, in *Desmogramma*, the rows are consistently well defined and composed of fine punctation. Nevertheless, these patterns vary among species, demonstrating that such characteristics may occur in both genera and therefore cannot be considered fully diagnostic.

Likewise, coloration should not be considered a diagnostic character, since in *Grammodesma* there is broad variation in color, as well as in the shape and distribution of the elytral markings, without a consistent pattern. In contrast, species of *Desmogramma* follow a more defined pattern, being typically black or brown, with only yellowish longitudinal stripes on the elytra, without maculation.

With respect to the presence of a “deep longitudinal depression” on the last abdominal segment of males, this feature should also not be taken into consideration, as females do not exhibit this characteristic. Therefore, when distinguishing genera within Chrysomelinae, neither coloration, punctation, nor the presence of a depression on ventrite V in males should be regarded as diagnostic for either *Desmogramma* or *Grammodesma*. Similar patterns associated with these characters may also occur in other genera of the subfamily, including *Elytrosphaera*, rendering them unreliable for distinguishing these taxa.

Therefore, the most reliable characters for distinguishing *Grammodesma* from *Desmogramma* are body size and the robustness of the prosternal process. In *Grammodesma*, specimens are generally larger (9.2–12.0 mm in length) and exhibit a more robust prosternal process, with the apex typically about 1.5 times wider than the base. In contrast, *Desmogramma* species are smaller (6.5–9.0 mm) and possess a less robust prosternal process, with the apex usually three times wider than the base. These two diagnostic characters were used by Sampaio & Fonseca (2023) in couplet 6(5) of their identification key to distinguish *Grammodesma* from *Desmogramma*.

In the same publication in which *Grammodesma* was described, Achard (1923) transferred *Chrysomela luridipennis* to this genus, arguing that the species corresponded more appropriately to *Grammodesma* than to *Chrysomela* Linnaeus, 1758, where it had previously been listed by Stål (1865), and also more correctly than to *Deuterocampta* Chevrolat, 1836, as suggested by several later authors. However, *G. luridipennis* does not exhibit the most diagnostic feature of *Grammodesma*: a robust prosternal process that is abruptly angled (with the basal portion projected and forming approximately a 90° angle in lateral view). For this reason, the species is here transferred to *Elytrosphaera*, the genus to which it was originally assigned, as it displays the typical and diagnostic morphological characters defining that taxon.

***Elytrosphaera*** **Blanchard 1845**

The genus was first mentioned in Dejean’s catalogue (1836), but it was only formally described by Blanchard (1845). In that work, the author proposed the following diagnostic characters for the genus: antennae filiform, robust; antennomeres subequal in length; elytra convex, ovoid, wider than pronotum; palpi with expanded apical segment. However, as also discussed for *Grammodesma*, the characters proposed by Blanchard (1845) are not effective for the identification of the genus, since they can likewise be observed in other Chrysomelinae taxa.

Another important source of nomenclatural confusion concerns the relationship between *Elytrosphaera* Blanchard 1845 and *Comisteisa* Gistel, 1848, particularly regarding the priority and synonymy of these names. Monrós & Bechyně (1956) mistakenly cited “Gistel 1837: 403” for *Comisteisa*, although this reference actually corresponds to Gistel (1848). Based on this incorrect date, they gave priority to *Comisteisa* over *Elytrosphaera* and consequently synonymized the two genera, leading Bechyně to subsequently place species formerly assigned to *Elytrosphaera* in *Comisteisa*. Conversely, Seeno & Wilcox (1982) and Daccordi (1994) listed *Comisteisa* as a synonym of *Elytrosphaera* Chevrolat, 1843. However, neither interpretation accurately reflects the nomenclatural history of these names.

Gistel (1848) attributed *Comisteisa xanthoptera* to Perty; however, no corresponding description by Perty has been located. Therefore, the authorship of the taxon should be attributed to Gistel, as there is no clear evidence that Perty provided the description. *Comisteisa xanthoptera* had appeared in three earlier publications, but only as a *nomen nudum*: Perty (1830: 18), as “*Chrysomela xanthoptera* Pty.”; Gistel (1834), as “*Doryphora xanthoptera* Pty.”; and Gistel (1846: 134), as “Nov. Gen. (*Chrys. xanthoptera* Perty)”. Consequently, *Elytrosphaera* Blanchard 1845 has priority over *Comisteisa* Gistel, 1848. Furthermore, because the type species of *Elytrosphaera* and *Comisteisa*, *E. xanthopyga* Stål, 1858 and *C. xanthoptera* Gistel, 1848, respectively, were synonymized by Monrós & Bechyně (1956), *Comisteisa* must be regarded as a junior synonym of *Elytrosphaera*, as thoroughly investigated and clarified by Bezděk (2020: 181).

In their identification key to the genera of Chrysomelinae recorded in Brazil, Sampaio & Fonseca (2023) included both *Elytrosphaera* and *Grammodesma*. *Elytrosphaera* is placed at couplet 30(28), whereas *Grammodesma* appears at couplet 6(5). Although their positions in the key do not provide evidence of phylogenetic relationships, the diagnostic characters used in the key are useful for distinguishing these genera. One of the relevant characters separating *Grammodesma* from *Elytrosphaera* is the non-projected base of the prosternal process, as indicated in couplet 5(4).

Thus, certain diagnostic characters can be established for *Elytrosphaera* within Chrysomelinae. In this group, the morphological combination of characters is fundamental for delimiting the genus, particularly with regard to the configuration of the ventral processes, as already emphasized by Sampaio & Fonseca (2023). Among the main attributes observed are: head with vertexal fovea; clypeus trapezoidal; genofrontal suture visible; prosternal process shallow, base not projected, apex with median depression; mesoventral process short, oblique; metaventral process as prominent as mesoventral. Pronotum with coarse, irregular punctation. Elytra with coarse punctation, mostly in series, irregular along margins and apex; outer margin strongly rounded; longitudinal bands present or absent.

Throughout the taxonomic history of *Elytrosphaera*, *Elytrosmena* Motschulsky, 1860 has been regarded as a closely related genus. It was initially treated as a subgenus of *Elytrosphaera* and was later elevated to generic rank by Daccordi (2008). *Elytrosmena* is a South American genus, predominantly Andean, with most of its species associated with humid montane environments. Many species exhibit restricted distributions, probably as a consequence of apterism, which limits dispersal and promotes endemism. The genus occurs in Argentina, Colombia, Ecuador, and Peru, and Daccordi (2008) mentioned a single record from Brazil (Santa Catarina) referring to *Elytrosmena fulminigera* Stål, 1858; however, this record is considered doubtful and possibly the result of a labeling error. Currently, *Elytrosmena* comprises 29 described species, with *Elytrosmena testudinaria* Stål, 1858 designated as the type species of the genus.

*Elytrosphaera*, previously regarded as the “true *Elytrosphaera*” (*Elytrosphaera* sensu stricto) prior to Daccordi (2008), was considered in the same work to comprise eight valid species and one subspecies: *E. breviuscula* Stål, 1858; *E. cupreata* Jacoby, 1903; *E. laeta* Bechyně, 1952; *E. lahtivirtai* Bechyně, 1951; *E. noverca* Stål, 1858 (= *E. vittata misionesa* Bechyně, 1950); *E. sulcipennis* Bechyně, 1950; *E. vittata vittata* Baly, 1858; and *E. x. xanthoptera*. Its known distribution is restricted to South America, occurring in Brazil (Southeastern and Southern regions) and Argentina (Misiones).

As observed in *Elytrosmena*, species of *Elytrosphaera* are apterous, a condition that results in a high degree of endemism and in occurrence patterns associated with humid Atlantic Forest environments within relatively small geographic areas. The inability to fly appears to limit the colonization of new habitats and reinforces the genus’s dependence on continuous and isolated forested environments. This relationship is supported by feeding records on species of Asteraceae and Solanaceae, indicating possible ecological specificity and further highlighting the vulnerability of the group to contemporary forest fragmentation.

The apterism documented in *Elytrosphaera* may represent an important factor influencing the biological vulnerability of the genus. By limiting flight-mediated dispersal, this condition may constrain range expansion and the recolonization of degraded areas. Such limitations may be particularly relevant for *Elytrosphaera*, since several species have highly localized distributions within the Atlantic Forest, and habitat destruction has previously been identified as a potential threat to some species (Macedo et al. 1998). Thus, although dispersal capacity and extinction risk were not directly evaluated here, the combination of apterism, restricted distributions, and ongoing habitat alteration suggests that some species of *Elytrosphaera* may be particularly vulnerable to habitat fragmentation and other anthropogenic disturbances.

*Elytrosphaera* has generally been treated by several authors as a completely apterous genus. However, a detailed analysis of its species reveals that *E. luridipennis* possesses small membranous wings with a reduced anal lobe. This pattern is consistent with the trends of wing reduction described by Jolivet (1959) for Chrysomeloidea, which include reduction of the anal field, simplification, fusion, or loss of the anal veins, as well as extreme reduction of the anal lobe, which may assume vestigial or lobiform shapes, particularly in apterous or micropterous lineages.

Jolivet (1957) further emphasizes that wing reduction is frequently associated with a sedentary lifestyle, insular or montane environments, as well as edaphic or cryptic habits. In extreme cases, according to the author, the wings may be reduced to lobiform rudiments with unrecognizable venation.

**Fig. 1:**
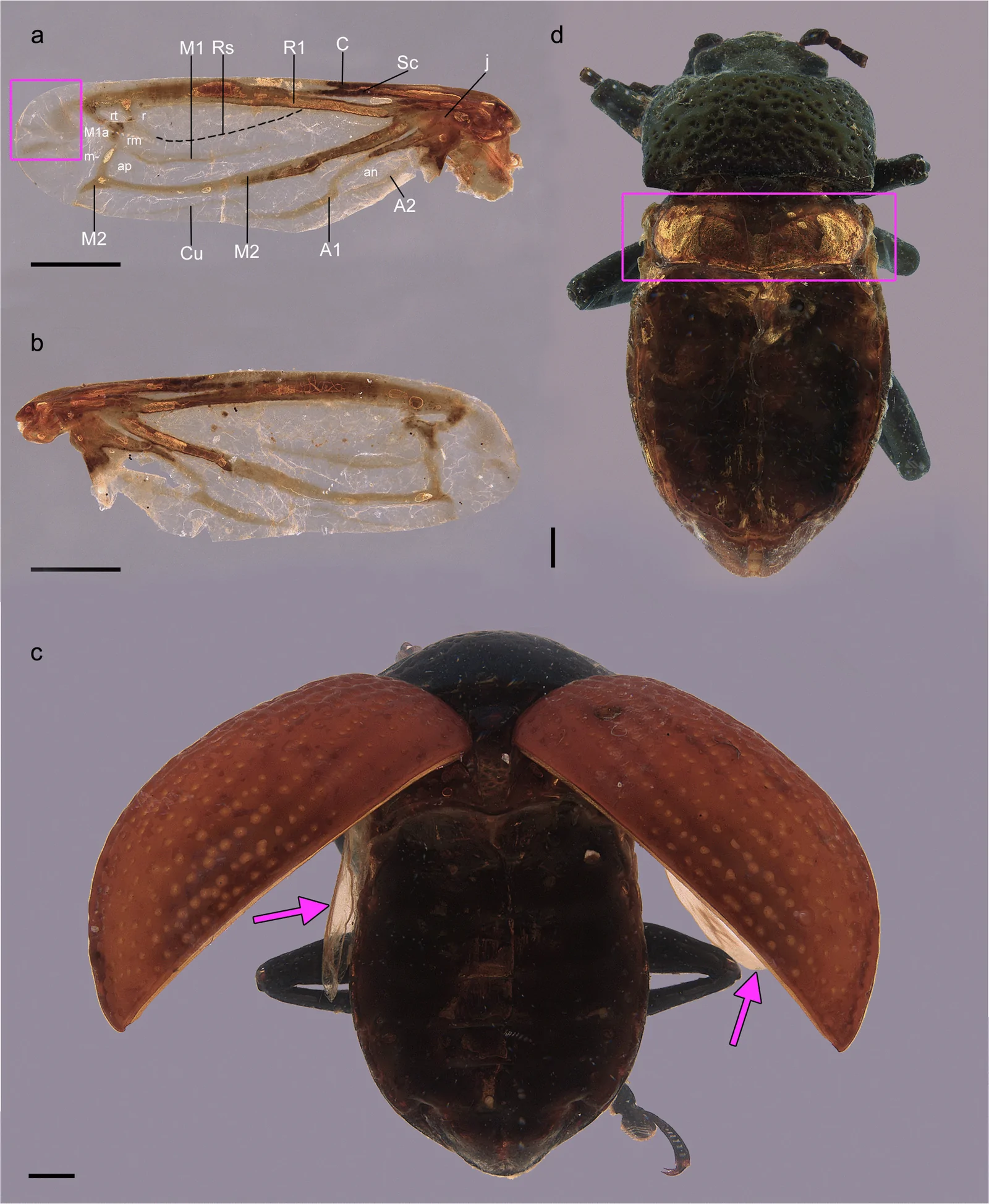
*Elytrosphaera* Blanchard 1845. (**a–c**) *Elytrosphaera luridipennis* Baly 1859: (**a**) left membranous wing; (**b**) right membranous wing; (**c**) membranous wings. (**d**) *Elytrosphaera xanthoptera xanthoptera* (Gistel, 1848): metathorax. Scale bar = 1 mm

The author also presents a theoretical Chrysomeloidea wing as a morphological reference, used for the interpretation and homologization of wing veins. Accordingly, based on the theoretical model proposed by Jolivet (1957), the wing veins of *E. luridipennis* were named (Figs. 1a, b, c). As noted by Jolivet, the distal region of the wing shows a strong reduction of longitudinal veins, which may persist only as faint traces or vestiges. In particular, the distal disappearance of the anterior median vein (M1) is recurrent in Chrysomeloidea, whereas the radial sector (Rs) is generally represented distally by incomplete, weakly sclerotized, or vestigial branches, often suggested only by slight chitinous thickenings and lacking a clear structural role.

Among the eight species currently recognized in *Elytrosphaera*, *E. luridipennis* stands out as one of the most morphologically distinct species within the genus. This distinction is mainly due to its status as the largest species in the group, in addition to presenting a set of relevant diagnostic characters.

*Elytrosphaera luridipennis* is distinguished by the following characters: clypeus usually with paired foveae; frontogenal suture invisible; posterior margin of frons with or without paired median foveae; prosternal process without basal fovea; and elytra dark reddish brown. The characters previously used to distinguish *E. lahtivirtai* from *E. luridipennis* were found to be variable among the specimens examined and do not provide consistent diagnostic differences between the two nominal species. In particular, the presence or absence of paired foveae on the clypeus and posterior margin of the frons varies among specimens, whereas the remaining diagnostic characters overlap between the two nominal taxa. Therefore, these differences are here interpreted as intraspecific variation rather than evidence of species-level separation. This interpretation is further supported by comparison of the original descriptions, examination of non-type specimens previously identified as *E. lahtivirtai* (including specimens identified by Jan Bechyně, the author of the species), and examination of photographs of the type specimens of both nominal species. Accordingly, *E. lahtivirtai* is here considered a junior synonym of *E. luridipennis*.

***Elytrosphaera luridipennis*** **Baly 1859**

(Figs. 1a–c, 2, 6, 3, 4, 5, 8, 9a–f, 12a–d)

**Fig. 2:**
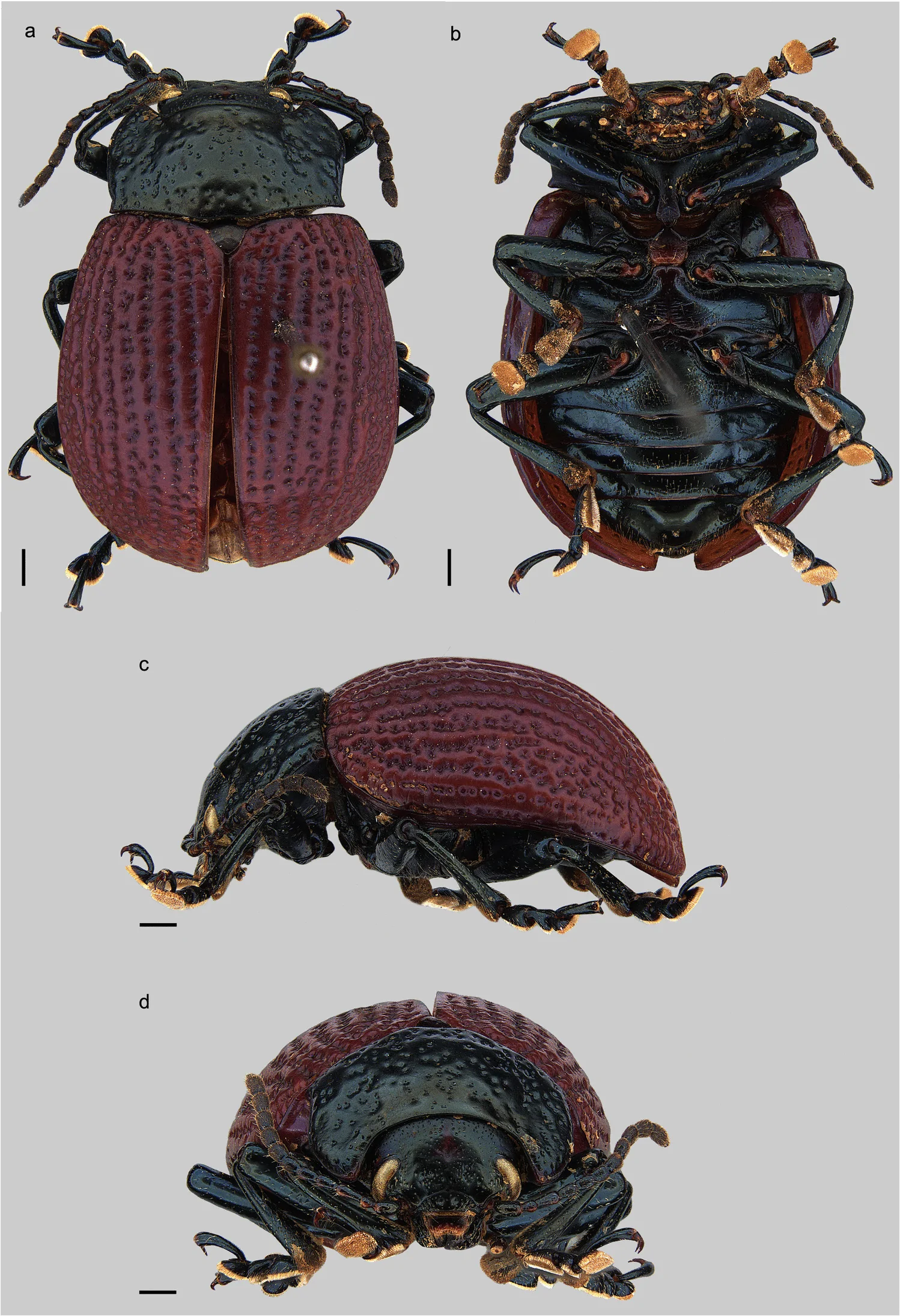
*Elytrosphaera luridipennis* Baly 1859. (**a**) dorsal view; (**b**) ventral view; (**c**) lateral view; (**d**) frontal view. Scale bar = 1 mm

*Elytrosphaera luridipennis* Baly 1859: 58 (original description).

*Chrysomela luridipennis*: Stål, 1865: 215 (distribution, redescription).

*Deuterocampta luridipennis*: Gemminger & Harold 1874: 3442 (catalog, distribution); Weise 1916: 49 (catalog, distribution); Blackwelder 1946: 676 (catalog, distribution); Bechyně, 1952: 37 (catalog, distribution).

*Grammodesma luridipennis*: Achard 1923: 72 (citation, taxonomy); Bechyně, 1952: 29 (catalog, distribution); Sampaio & Monné, 2016: 58, 59 (inventory, key); Flinte et al. 2017: 9 (checklist, distribution); Sampaio & Sekerka, 2025 (online checklist).

*Elytrosphaera lahtivirtai* Bechyně, 1951: 64 (original description, distribution, taxonomy), Fig. 1 (dorsal view); Zikán & Zikán, 1967: 129 (checklist, distribution); Jolivet & Vasconcellos-Neto 1993: 322 (biology, distribution, ecology); Macedo et al. 1998: 271–273, 275, 276 (biology, citation, distribution, host plant, taxonomy), Fig. 1D (dorsal view), 3 A (habitat), 3B (larva on host plant), 3 C (adult on host plant); Vasconcellos-Neto & Jolivet 1998: 300–302, 304, 305, 307 (biology, distribution, ecology, host plant), tabelas 1 (frequency of specimens collected per century), 2 (frequency of specimens in each month of the year); Daccordi 2008: 420 (citation); Bergallo et al. 2009: 145 (biology, citation, distribution); Flinte et al. 2017: 9 (checklist, distribution); Sekerka & Sampaio 2025 (checklist). **Syn. nov**.

*Elytrosphaera* (*Elytrosphaera*) *lahtivirtai* Bechyně, 1952: 39 (catalog, distribution).

**Type locality.** Brazil.

**Fig. 3:**
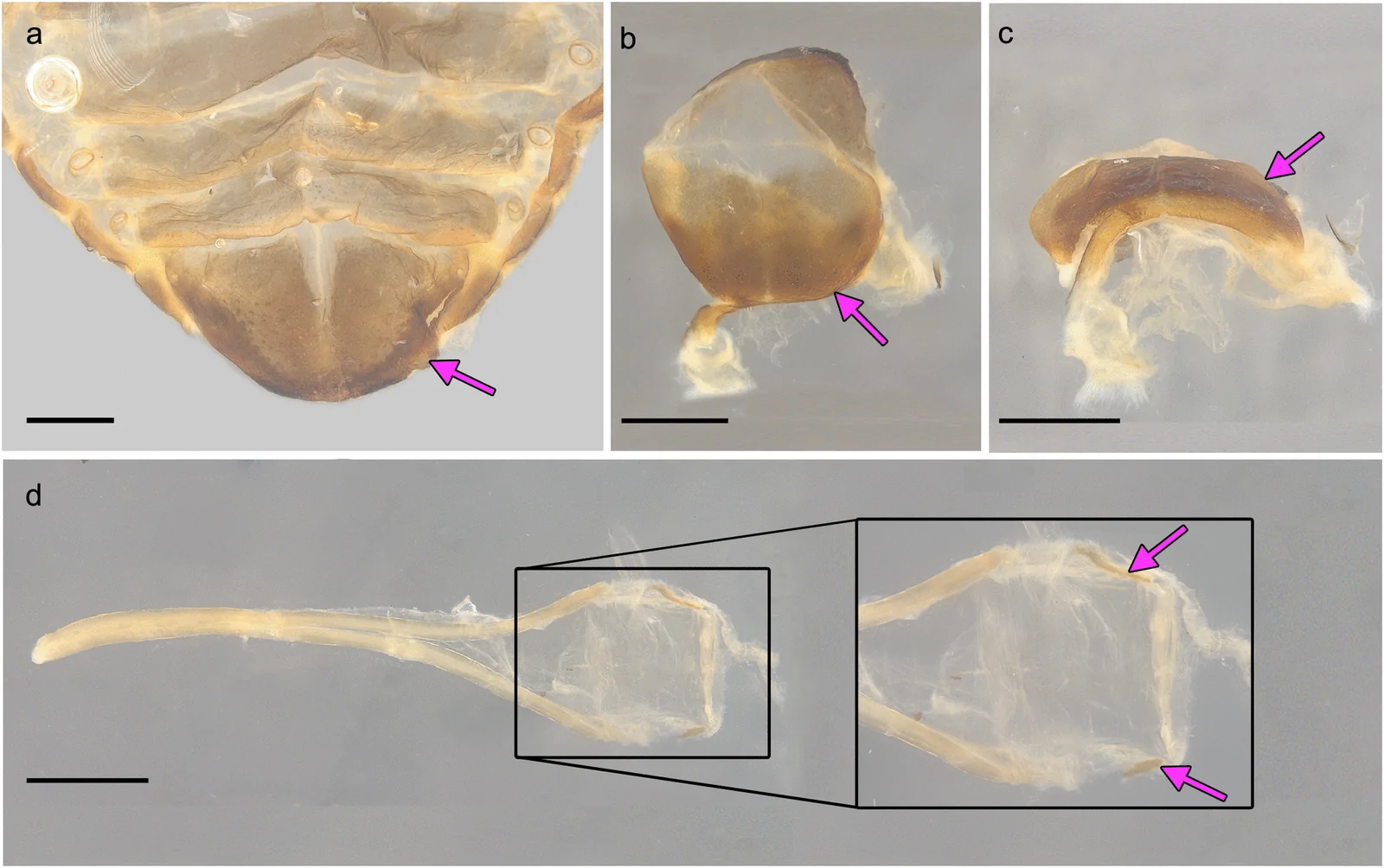
Male of *Elytrosphaera luridipennis* Baly 1859. (**a**) pygidium; (**b**,** c**) tergite IX; (**d**) *spiculum gastrale* and apical sclerites. Scale bar = 1 mm

**Redescription.** Body elongate; length 10.7–14.2 mm (clypeus–elytral apex); width 7.1–9.3 mm (at mid-elytra). ***Coloration*** (Figs. 2, 8a, b, d–f) black and reddish-brown, with slight greenish metallic reflections ventrally. ***Head*** (Figs. 2d, 12a) finely to coarsely punctate; vertex with fovea. Eyes oblong. Pedicel globose. Antennomeres III–V with sparse setae; VI–XI robust, densely pubescent. Antennomere III 1.2–1.3 times longer than IV. Prementum robust, convex. Labial palpomere III subcylindrical, apex truncate. Mentum with lateral margins slightly prominent; median region with fovea or constriction. Clypeus subtrapezoidal, with or without a pair of foveae. Frontogenal suture not visible. Supraocular suture visible or not. Frontal suture weakly defined. Posterior margin of frons with or without a pair of median foveae. Labrum apically emarginate, with long, sparse setae. Mandibles robust, with long, sparse setae. Maxillary palpomere III subconical, 1.8–2.1 times longer than IV; IV apically truncate. ***Prothorax*** (Figs. 2a, 8a, d) with lateral margins slightly rounded. Pronotum densely and coarsely punctate; punctures irregular. Prosternal process without basal fovea; apex with median depression. Mesoventral process short, slightly inclined, usually without median longitudinal depression; lateral margins not dilated. Metaventrite with convex surface; with long, sparse setae; anterior margin strongly margined except medially; process subtrapezoidal. Scutellum subtriangular. ***Elytra*** (Figs. 2a–c, 8a, b, d, e) rounded; posterior third slightly elongate; punctation coarse, partly arranged as rows; external margin rounded. Epipleura glabrous. ***Legs*** (Figs. 2b, 8b, e) robust, large; punctation fine, sparse; setae short, sparse; tibial apex densely pubescent. Coxae and tibiae elongate. Metatarsomere I strongly elongate. Tarsomere V without ventroapical projection, usually with two or more pairs of long setae. Claws simple. ***Abdomen*** (Figs. 2b, 6, 3a, 5a, b, 8b, e) finely and sparsely punctate; median region with abundant long setae. Process slightly medially emarginate at apical margin. Pygidium with or without median longitudinal groove. Pygidium–tergite IX connective membrane sclerotized. Ventrite V apical margin truncate or rounded.

**Fig. 4:**
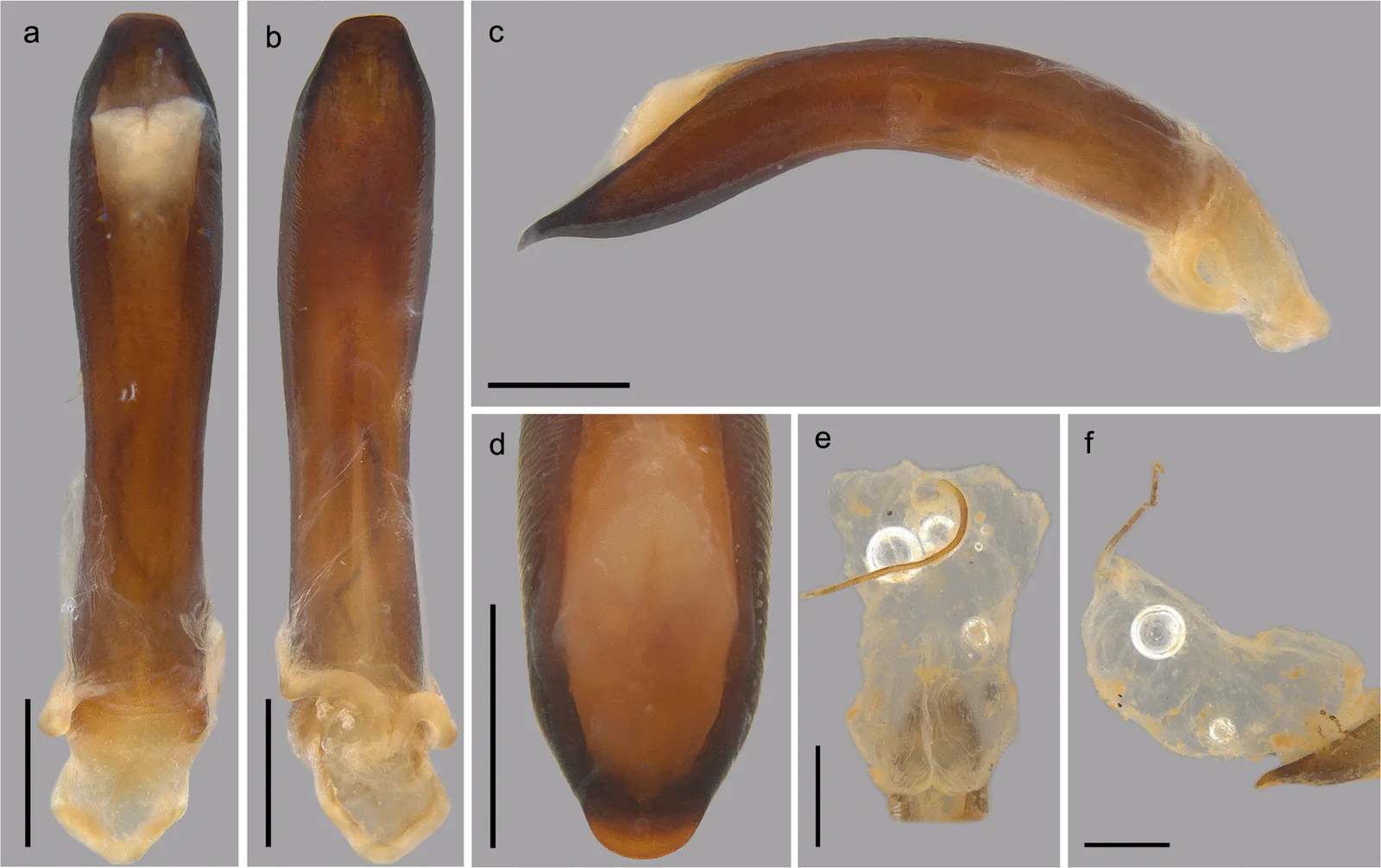
Aedeagus of *Elytrosphaera luridipennis* Baly 1859. (**a**) dorsal view; (**b**) ventral view; (**c**) lateral view; (**d**) apex; (**e**, **f**) endophallus. Scale bar = 1 mm

**Fig. 5:**
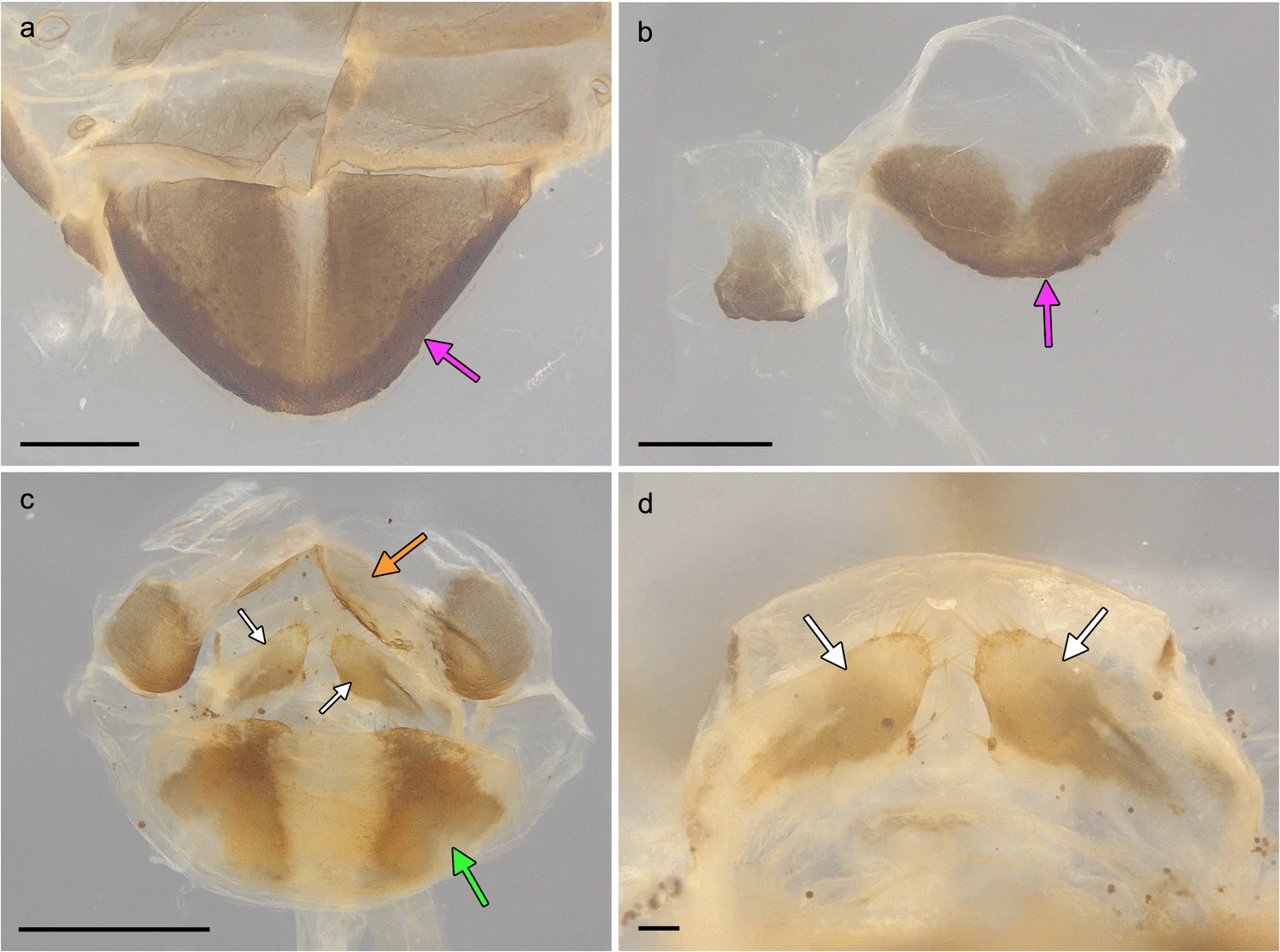
Female of *Elytrosphaera luridipennis* Baly 1859. (**a**) pygidium; (**b**) sclerotized connective membrane pygidium–tergite IX; (**c**) female genitalia: tergite IX (orange arrow), vaginal palps (white arrows), sternite IX (green arrow); (**d**) vaginal palps. Scale bars = 1 mm (a–c), 0.1 mm (d)

**Description. *Male genitalia*** (Figs. 3b–d, 4)**:** Tegmen slightly sclerotized, extending beyond basal lobe; base V-shaped; apices reaching or not the lateral margin of dorsal region of basal lobe. Aedeagus symmetrical, elongate, sclerotized, without lateral constriction between basal and median lobes; basal lobe elongate, apex as wide as base, apical tubercles inconspicuous; median lobe unarmed, slightly widened in apical half, apical extremity rounded, slightly curved ventrally, ventral region concave (more evident in lateral view); ligula inconspicuous; ostium longer than wide; endophallus membranous, without sclerites, with filiform flagellum. *Spiculum gastrale* moderately sclerotized, Y-shaped, with two elongate apical sclerites, one on each side; bases united, not fused; length subequal to aedeagus. Tergite IX convex, margins ventrally curved; apical margin with abundant setae. ***Female genitalia*** (Figs. 5c, d)**:** Tergite IX large, V-shaped, with dorsal longitudinal groove; apical margin rounded, internally curved, medially emarginate, with long setae. Vaginal palpi broad, spatulate, sclerotized, elongate; base slightly membranous; apex gently rounded, with long and short setae on apical and inner margins; with two accessory basal sclerites (one per side). Sternite IX broad, irregular in shape, medially weakly sclerotized; apical margin curved upward, with setae. Spermathecal duct and spermatheca absent.

**Geographical distribution.** BRAZIL: *Rio de Janeiro* (Itatiaia); *São Paulo* (Cantareira). ***New records:**** Espírito Santo* (Castelo; Vargem Alta); *Minas Gerais* (Fazenda dos Campos; Passa Quatro); *Rio de Janeiro* (Nova Friburgo; Resende); *São Paulo* (Perequê).

**Host plant.** *Solanum caeruleum* Vell. (Solanaceae) (Marcedo et al. 1998).

**Fig. 6:**
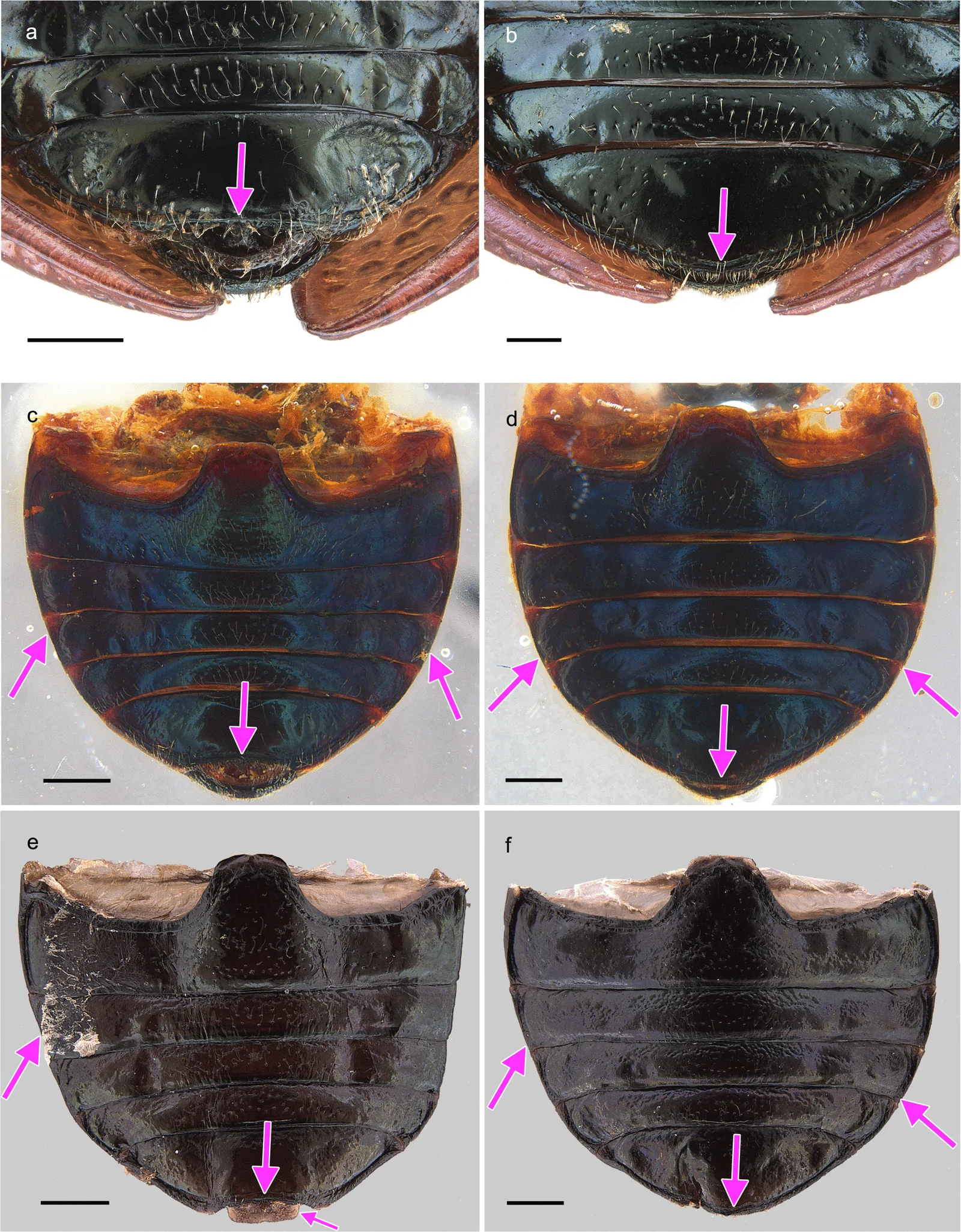
Abdomen of *Elytrosphaera luridipennis* Baly 1859. (**a**,** c**,** e**) Male; (**b**,** d**,** f**) female. (**a**,** b**) apical margin ventrite V; (**c**,** d**) lateral margins abdomen and apical margin ventrite V; (**e**,** f**) lateral margins abdomen, apical margin ventrite V, and sclerotized membrane inner surface ventrite V. Scale bar = 1 mm

**Sexual dimorphism** (Fig. 6)**.** *Male*: abdomen slightly wider, lateral margins weakly rounded; ventrite V apically truncated, with sclerotized inner apical membrane. *Female*: abdomen narrower, lateral margins strongly rounded; ventrite V apically rounded, without sclerotized inner apical membrane.

**Fig. 7:**
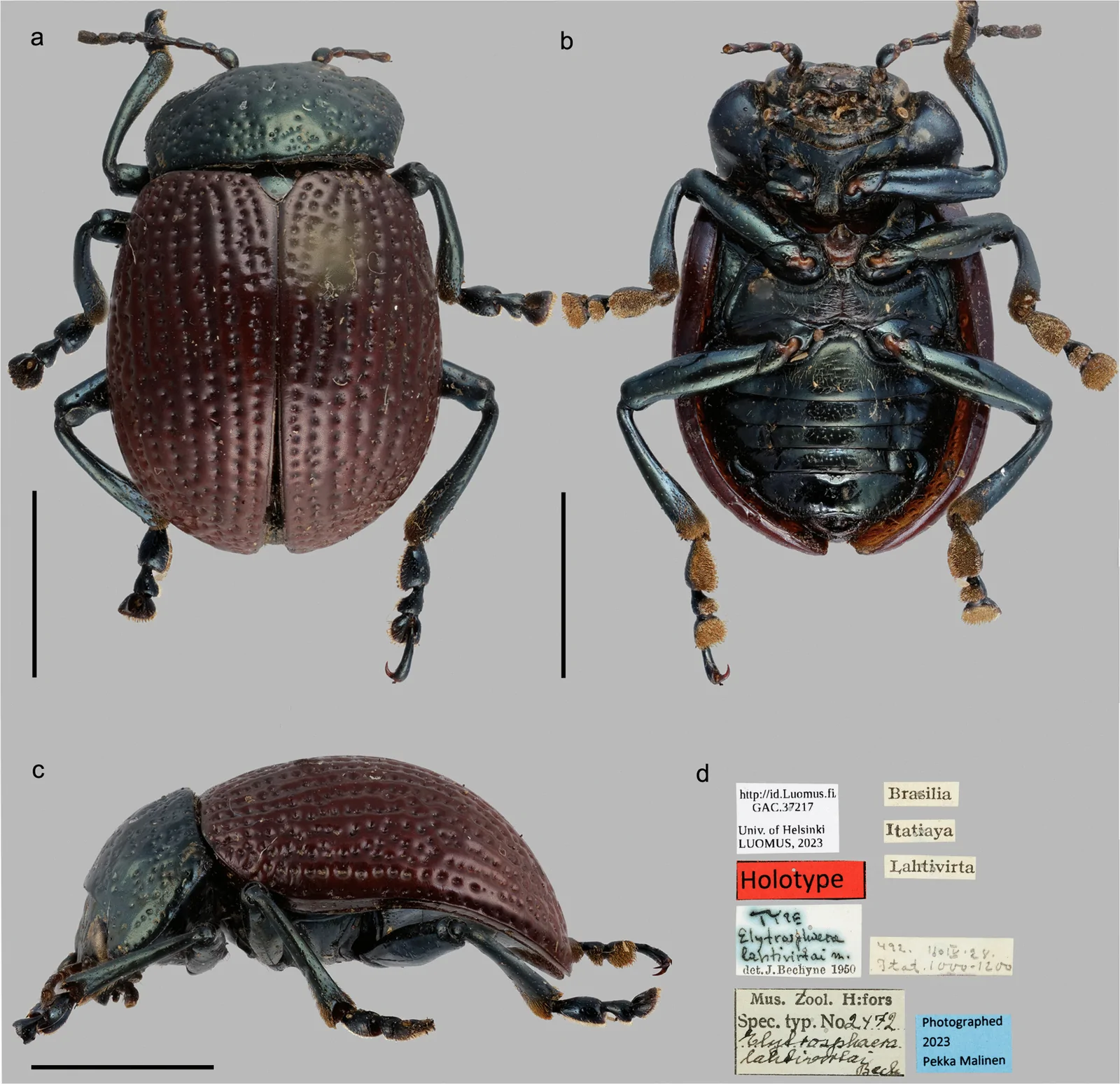
Holotype of *Elytrosphaera lahtivirtai* Bechyně, 1951. (**a**) dorsal view; (**b**) ventral view; (**c**) lateral view; (**d**) labels. Scale bar = 5 mm. Photographed by Pekka Malinen (© 2023 Finnish Museum of Natural History, University of Helsinki). Original photos cropped

**Fig. 8:**
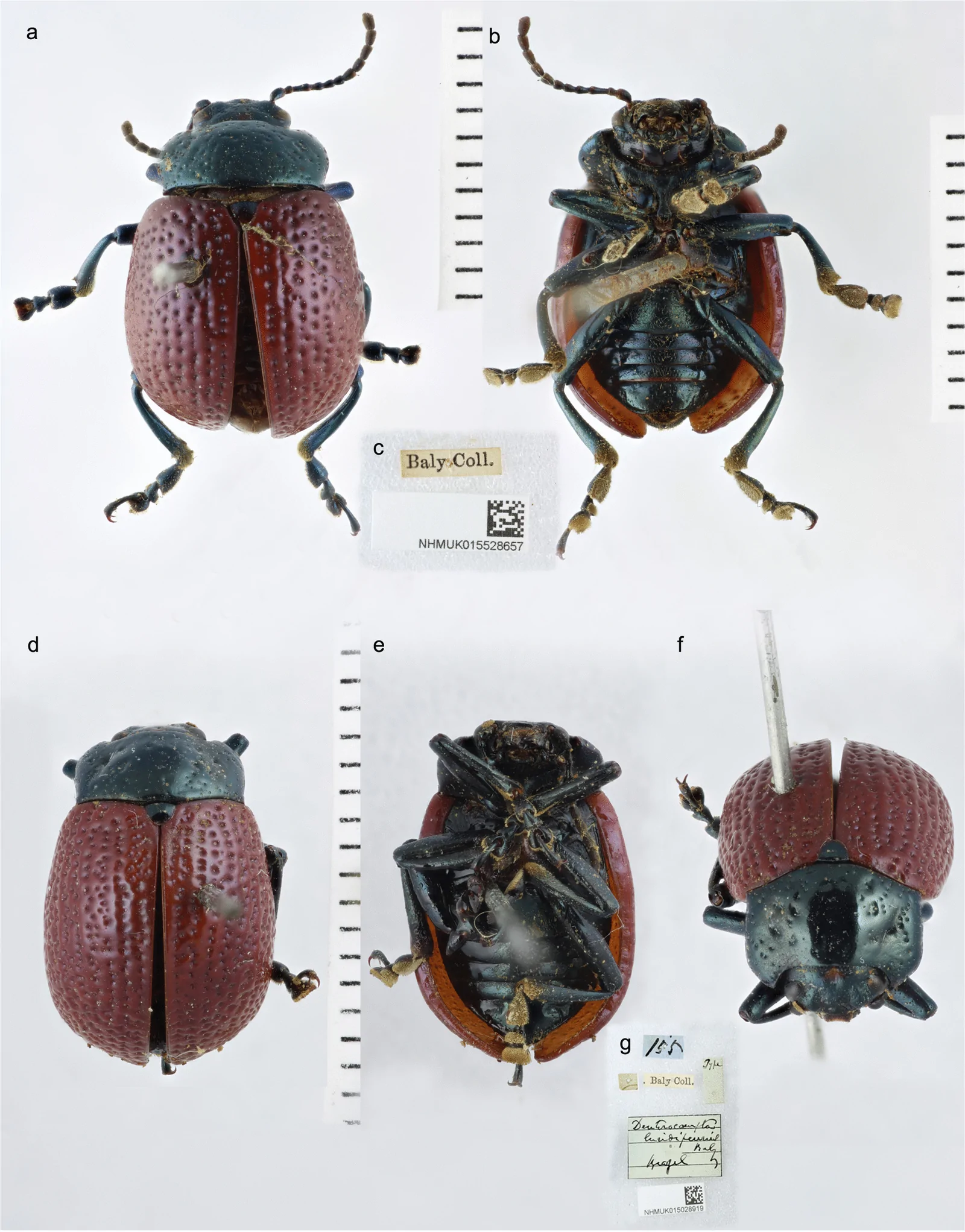
Syntypes *Elytrosphaera luridipennis* Baly 1859. (**a**, **d**) dorsal view; (**b**, **e**) ventral view; (**f**) frontal view; (**c**, **g**) labels. Photographed by Keita Matsumoto (© 2023 Natural History Museum London). Original photos cropped

**Type material. *Elytrosphaera lahtivirtai*** Bechyně, 1951**: HOLOTYPE:** ♂ (Fig. 7), LUOMUS; bearing labels “Brasilia”, “Itatiaya”, “Lahtivirta”, “492. 11.IV.28./Itat. 1000–1200”, “TYPE/*Elytrosphaera/lahtivirtai* m./det. J. Bechyně 1950”, “Mus. Zool. H:fors/Spec. typ. No 2472/*Elytrosphaera/lahtivirtai*/Bech”, “http://id.luomus.fi/GAC.37217/Univ. of Helsinki/LUOMUS, 2023”, “Holotype”, “Photographed/2023/Pekka Malinen”. ***Elytrosphaera luridipennis*** Baly 1859**: SYNTYPES:** ♂ (Fig. 8a–c), NHMUK; bearing labels “Baly.Coll.”, “NHMUK015528657”; ♀ (Fig. 8d–g), NHMUK; bearing labels “155”, “Type”, “Baly.Coll.”, “*Deuterocampta*/*luridipennis* Baly/Brazil”, “NHMUK015028919”.

**Non-type material** (35♂♂, 29♀♀)**.** BRAZIL. Amazonas: *Benjamin Constant*, Rio Javari, 1♀, VII.1960, Dirings leg. (MZSP-68225). Minas Gerais: *Fazenda dos Campos*, 1♂, 10.XII.1915, J.F.Zikán leg. (CEIOC-28288); 1♀, 31XII.1922, J.F.Zikán leg. (CEIOC-28314); *Passa Quatro*, Serra dos Cochos, 1♂, 27.I.1923, J.F.Zikán leg. (CEIOC-28301); 1♂ e 1♀, 17.II.1923, 1460 m, J.F.Zikán leg. (CEIOC-28318); 1♂, 19.II.1923, J.F.Zikán leg. (CEIOC-28313); 2♂ e 1♀, 04.III.1923, J.F.Zikán leg. (CEIOC-28283); 1♂, 27.IV.1923, 850 m, J.F.Zikán leg. (CEIOC-28303). Rio de Janeiro: *Itatiaia*, 4♀, I.1925, J.F.Zikán leg. (CEIOC-28280); 1♂ e 1♀, XII.1925, J.F.Zikán leg. (CEIOC-28285); 1♀, II.1926, J.F.Zikán leg. (CEIOC-28306); 1♀, 21.XI.1932, J.F.Zikán leg. (CEIOC-28289); 1♂, I.1933, J.F.Zikán leg. (CEIOC-32896); 1♂, XII.1933, Dirings leg. (MZSP-68318); 1♂ e 2♀, 29.XII.1952 (Maromba), C. Leite, Seabra & Zikán leg. (CEIOC-28284); 1♂ e 2♀, I.1955, 1100 m, Dirings leg. (MZSP-68321, 68,325, 68,327); 1♂, I.1957 (P.N.do Itatiaia), L.C.Alvarenga leg. (DZUP-609844); 1♂, 20.XII.1957, 2648, A.P.da Silva leg. (MZSP-68323); 1♂, I.1961, Dirings leg. (MZSP-68241); 1♂, X.1961, 1100 m, Dirings leg. (MZSP-48220); 4♂ e 1♀, II.1966, Dirings leg. (MZSP-48221, 68,316, 68,322, 68,324, 68,326); 2♀, XI.1966, Dirings leg. (MZSP-68159, 68,274); 7♂ e 6♀, I.1967, Dirings leg. (MZSP-68157, 68,173, 68,176, 68,177, 68,244, 68,290, 68,291, 68,292, 68,293, 68,306, 68,308, 68,311, 68,312); 1♀, IX.1967 (P.N.Itatiaia), M. Alvarenga leg. (DZUP-609845); 2♂ e 1♀, II.1969, Dirings leg. (MZSP-68276, 68,278, 68,281); *Nova Friburgo*, Mury, 1♀, 27.XII.1980, Gred & Guimarães leg. (MZSP-68175); 1♂, 15.XII.1991, A.Bello leg. (CEIOC-28281); *Resende*, Visconde de Mauá, 1♂, 10.XII.1991, [without collector] (CEIOC-28316). São Paulo: *Cantareira*, 2♂ e 1♀, XII.1932, Dirings leg. (MZSP-68317, 68,319, 68,320); 1♂ e 1♀, XII. 1936, B. Pohl leg. (MIZA-0040522, 0040523); *Perequê*, 1♂, 1939, J.F.Zikán leg. (CEIOC-28302).

**Note.** According to the examined material, one specimen from MZSP shows a distribution distinct from that observed for the remaining specimens and from that expected for the occurrence of *Elytrosphaera*. The specimen recorded from Amazonas (Benjamin Constant, Javari River) most likely represents a labeling error, since the genus is otherwise known from the Atlantic Forest and has not been recorded from the Amazon biome. Therefore, this record was not considered a valid new occurrence. Additionally, regarding the presence of a sclerotized membrane on the inner portion of the apical margin of ventrite V, this structure is herein interpreted as a stiffened membrane, although its origin remains unknown, and it cannot be determined whether or not it corresponds to a true segment, a question that would require more detailed studies of the internal morphology of the group.

**Comment.** Many species belonging to different genera of Chrysomelinae exhibit similar color patterns, which may occasionally result in misidentifications. For this reason, the analysis of diagnostic structures is essential for accurate generic delimitation within the subfamily, especially those related to the combination of ventral processes, as mentioned above. In the context of these chromatic similarities among species belonging to distinct genera, *E. luridipennis* shows a coloration very similar to that of *G. rubroaenea*, originally described in *Deuterocampta*, although both can be clearly distinguished by the characters presented in Table 1.

**Table 1:** External morphological differences between *Elytrosphaera luridipennis* Baly 1859 and *Grammodesma rubroaenea* (Stål, 1859)

E. luridipennis	G. rubroaenea
Pedicel globose (Fig. 9a)	Pedicel elongate (Fig. 9g)
Antennomeres VII–X longer than wide (Fig. 9b)	Antennomeres VII–X as long as wide (Fig. 9h)
Prothorax lateral margins strongly rounded (Fig. 9c)	Prothorax lateral margins substraight (Fig. 9i)
Pronotum punctation coarse (Fig. 9c)	Pronotum punctation fine (Fig. 9i)
Scutellum wider than long, margins strongly rounded (Fig. 9d)	Scutellum longer than wide, margins straight (Fig. 9i)
Elytra posterior third slightly elongated (Fig. 9e)	Elytra posterior third strongly elongated (Fig. 9j)
Prosternal process base not projected (Fig. 9f)	Prosternal process base projected (Fig. 9k)
Mesoventral process oblique (Fig. 9f)	Mesoventral process straight (Fig. 9k)
Metaventral process surface convex (Fig. 9f)	Metaventral process surface nearly flat (Fig. 9k)
Metaventral process slender (Fig. 9f)	Metaventral process robust (Fig. 9k)

**Fig. 9:**
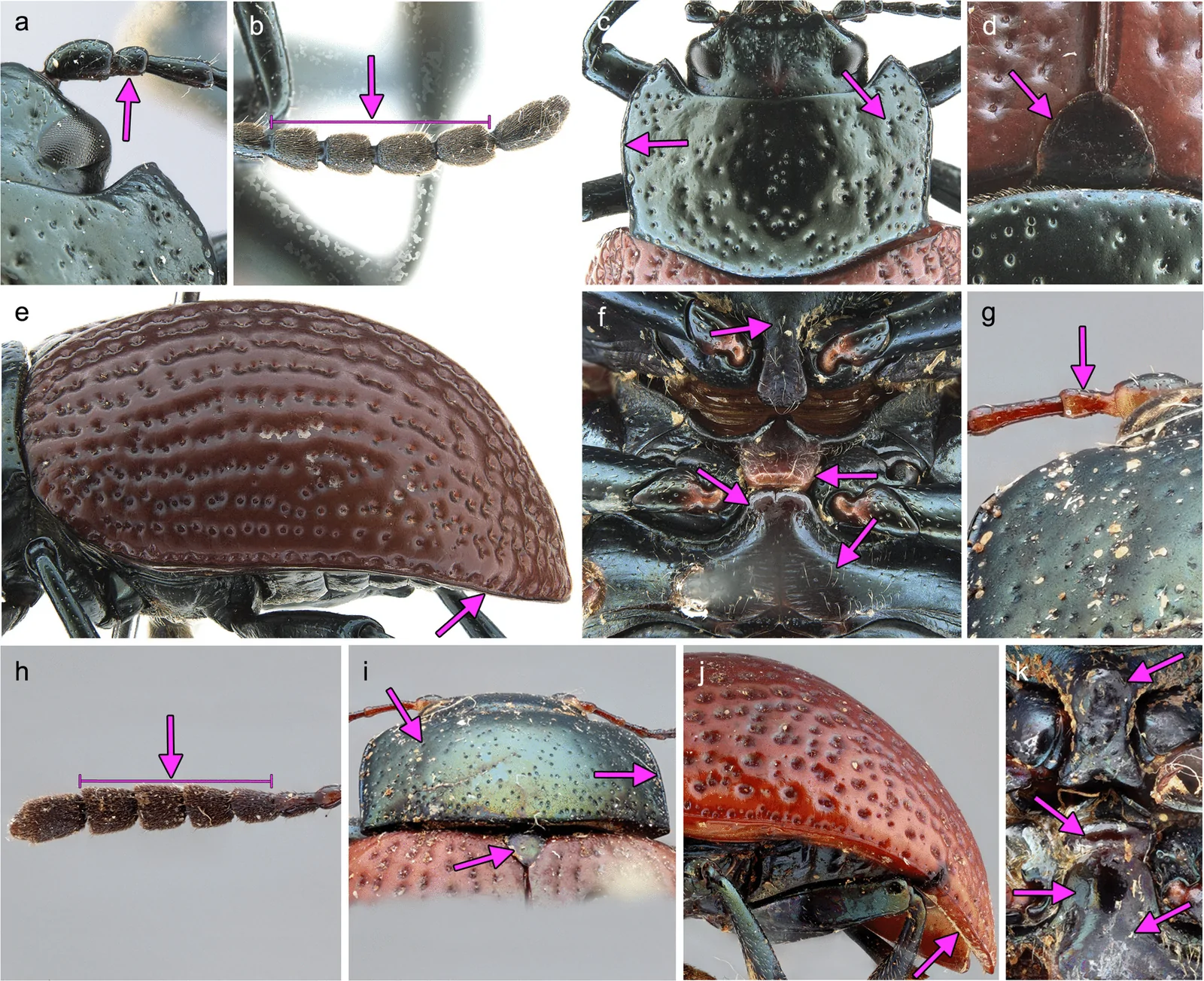
*Elytrosphaera* Blanchard 1845 and *Grammodesma* Achard 1923. (**a–f**) *Elytrosphaera luridipennis* Baly 1859; (**g–k**) *Grammodesma rubroaenea* (Stål, 1859). (**a**, **g**) pedicel; (**b**, **h**) antennomeres VII–X; (**c**) prothoracic lateral margins and pronotal punctation; (**d**) scutellum; (**e**, **j**) posterior third of elytra; (**f**, **k**) prosternal, mesoventral, and metaventral processes and metaventrite surface; (**i**) prothoracic lateral margins, pronotal punctation, and scutellum

**Fig. 10:**
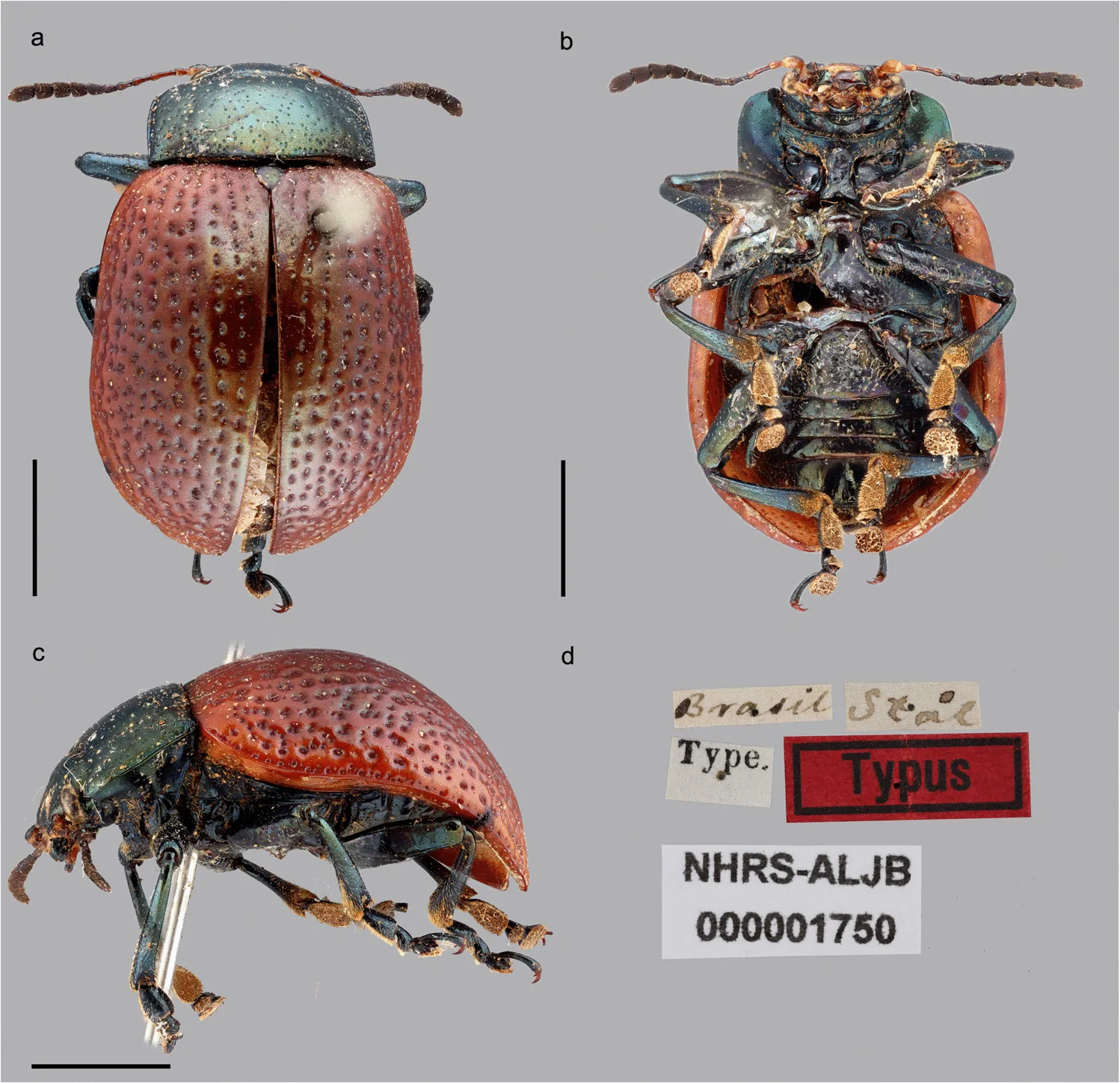
Syntype of *Grammodesma rubroaenea* (Stål, 1859). (**a**) dorsal view; (**b**) ventral view; (**c**) lateral view; (**d**) labels. Scale bar = 3 mm. Photographed by Anna Jerve (© 2023 Naturhistoriska Riksmuseet). Original photos cropped

**Comparative type material examined.** *Grammodesma rubroaenea* (Stål, 1859): **SYNTYPE:** ♂ (Fig. 10), NHRS; bearing labels “Brazil”, “Stål”, “Type”, “Typus”, “NHRS-ALJB 000001750”.

**Fig. 11:**
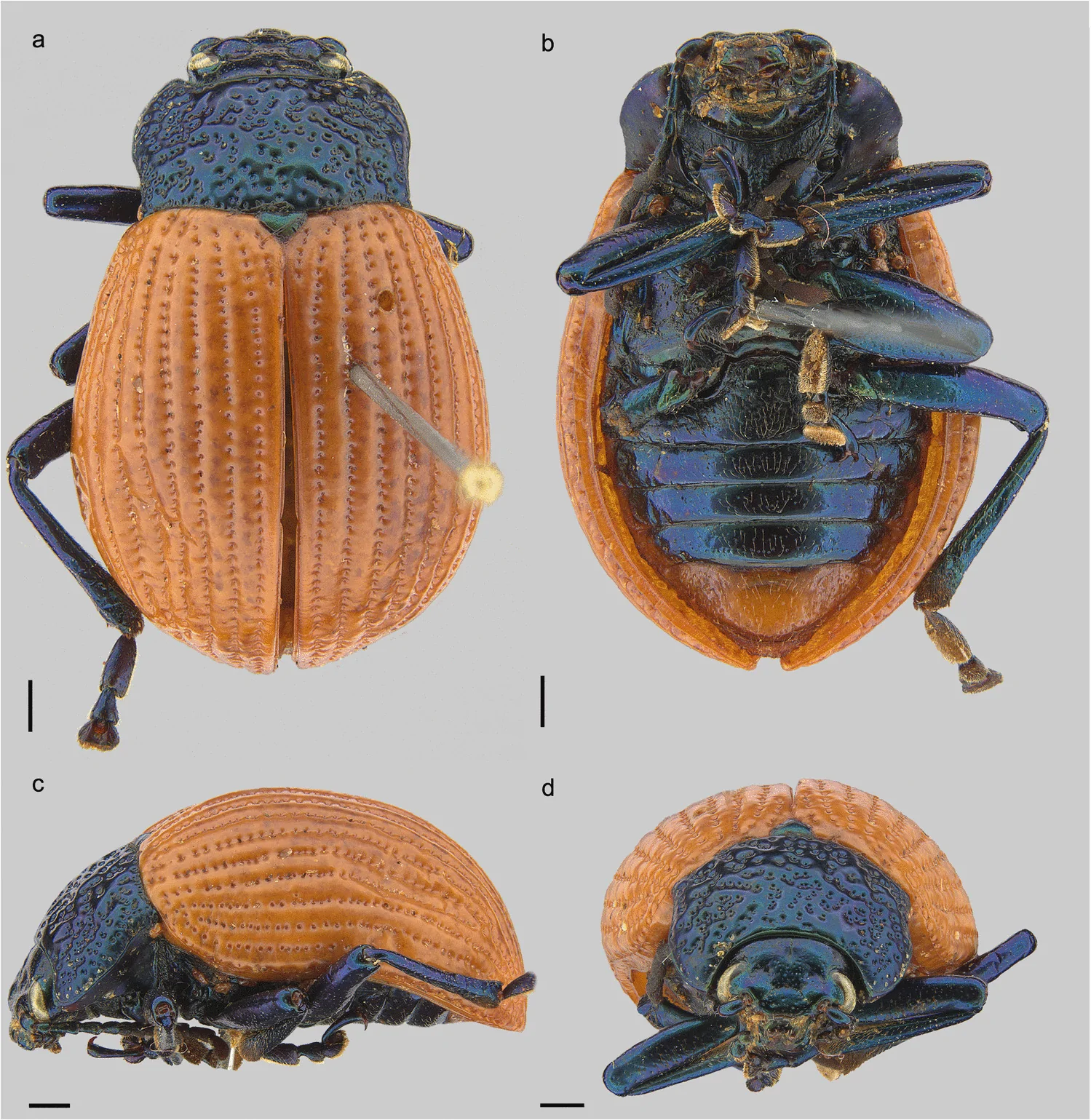
*Elytrosphaera xanthoptera xanthoptera* (Gistel, 1848). (**a**) dorsal view; (**b**) ventral view; (**c**) lateral view; (**d**) frontal view. Scale bar = 1 mm

Another relevant point concerns the comparison made by Bechyně (1951) when describing *E. lahtivirtai*. He compared this species with *Elytrosphaera xanthopyga* Stål, 1858, currently regarded as a synonym of *E. x. xanthoptera* (Figs. 1d, 11). Bechyně (1951) distinguished *E. lahtivirtai* from *E. xanthopyga* based on the following characters: *E. lahtivirtai*, body length 13.5–15 mm; head finely punctate; pronotum rather opaque, with well-developed microscopic reticulation (visible at 30 ×); prothorax strongly transverse, > 2 × wider than long, sides strongly rounded; elytra with irregular punctation, directed laterally and especially posteriorly; lateral elytral margins clearly visible dorsally (elytra not enclosing abdomen); male with fifth abdominal ventrite posteriorly truncate and tibiae strongly dilated. *E. xanthopyga*, body length 7–10.5 mm; head entirely smooth; pronotum very shiny, microscopic reticulation almost imperceptible (60 ×); prothorax weakly transverse, clearly < 2 × wider than long, sides slightly rounded; elytra with very regular punctation; lateral elytral margins not visible dorsally (elytra enclosing abdomen); male with fifth abdominal ventrite apically acuminate and tibiae only slightly dilated toward apex.

Among the characters proposed by Bechyně (1951), particular attention should be given to the statement that *E. xanthopyga* has an entirely smooth head. However, specimens of this taxon exhibit cephalic punctation, although very fine, as also observed in *E. luridipennis*. With regard to the strong dilation of the tibiae attributed by Bechyně (1951) to *E. lahtivirtai*, specimens of *E. luridipennis* are the largest and most robust within the genus *Elytrosphaera*, which naturally results in tibiae proportionally larger than those of *E. xanthopyga*. Nevertheless, tibiae slightly broadened apically are not exclusive to *E. luridipennis* and therefore do not represent a truly diagnostic feature for distinguishing these taxa. Although *E. x. xanthoptera* was considered by Bechyně (1951) to be the species morphologically most similar to *E. lahtivirtai*, our results show that *E. x. xanthoptera* and *E. luridipennis* exhibit several characters that clearly differentiate them, as summarized in Table 2.

**Table 2:** External morphological differences between *Elytrosphaera luridipennis* Baly 1859 and *Elytrosphaera xanthoptera xanthoptera* (Gistel, 1848)

E. luridipennis	E. x. xanthoptera
Body black, reddish brown, and/or dark brown (Figs. 2, 7a, b, c)	Body metallic blue to violet (Fig. 11)
Vertex with light- to reddish-brown spot (Fig. 12a)	Vertex without spot (Fig. 12e)
Frons posteriorly with paired medial foveae (Fig. 12c)	Frons with medial foveae (Fig. 12g)
Postclypeus usually with a pair of foveae (Fig. 12c)	Postclypeus with foveae (Fig. 12g)
Antennomere 1.8–2.0 × longer than wide (Fig. 12b)	Antennomere XI 2.4–2.9 × longer than wide (Fig. 12f)
Clypeus 1.2–2.0 × wider than long (Fig. 12c)	Clypeus 1.1–1.4 × wider than long (Fig. 12g)
Postclypeus posterior margin not projected (Fig. 12a)	Postclypeus posterior margin projected (Fig. 12e)
Elytra light to dark reddish brown (Figs. 2a, 3a)	Elytra yellowish to light brown (Fig. 11a)
Elytra with rounded apex (Fig. 12d)	Elytra with tapered apex (Fig. 12h)
Ventrite V black to dark brown (Fig. 2b)	Ventrite V usually yellowish (Fig. 11b)

**Fig. 12:**
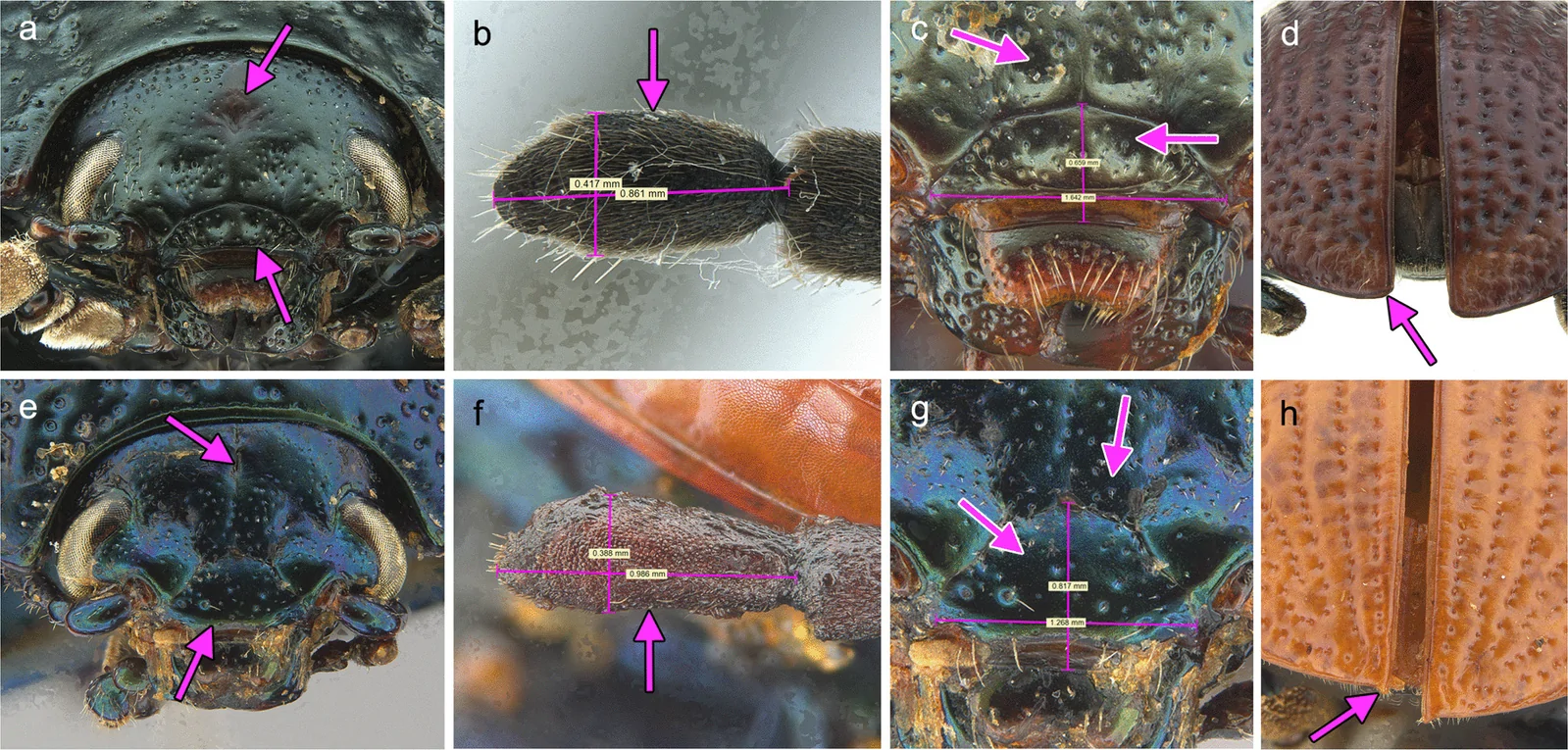
*Elytrosphaera* Blanchard 1845. (**a–d**) *Elytrosphaera luridipennis* Baly 1859; (**e–h**) *Elytrosphaera xanthoptera xanthoptera* (Gistel, 1848). (**a**, **e**) vertex and clypeus; (**b**, **f**) antennomere XI; (**c**, **g**) frons and clypeus; (**d**, **h**) elytral apex

## Data Availability

All data supporting the findings of this study are included in the article.
